# Impact of hand grip strength reduce on health related quality of life in patients with transfusion-dependent thalassemia insights from the SF-36 survey: a cross-sectional analysis

**DOI:** 10.3389/fmed.2025.1657096

**Published:** 2025-09-22

**Authors:** Mustafa Duran, Nermin Keni Begendi, Sinan Kazan, Hamza Sümter, Nigar Abdullayeva, Yusuf Ulusoy, Mehmet Enes Süzek, Nur Akad Soyer

**Affiliations:** ^1^Division of Hematology, Department of Internal Medicine, School of Medicine, Afyonkarahisar Health Science University, Afyonkarahisar, Türkiye; ^2^Division of Nephrology, Department of Internal Medicine, School of Medicine, Afyonkarahisar Health Science University, Afyonkarahisar, Türkiye; ^3^Istanbul Training and Research Hospital, Istanbul, Türkiye; ^4^Department of Hematology, Faculty of Medicine, Ege University, Izmir, Türkiye; ^5^Department of Hematology, Başakşehir Çam and Sakura City Hospital, Istanbul, Türkiye; ^6^Department of Internal Medicine, School of Medicine, Afyonkarahisar Health Science University, Afyonkarahisar, Türkiye

**Keywords:** transfusion-dependent thalassemia, SF-36, muscle strength, iron overload, anemia

## Abstract

**Purpose:**

Thalassemias negatively impact health-related quality of life (HQoL) due to chronic anemia and complications from regular transfusions. This study aimed to investigate the effects of hand grip strength loss on QoL in patients with transfusion-dependent thalassemia (TDT) via the Short Form-36 (SF-36) health survey.

**Patients and methods:**

A cross-sectional study included 47 patients with transfusion-dependent thalassemia (TDT) and a group of healthy controls. Hand grip strength was assessed via a digital handgrip dynamometer. HQoL was evaluated through the SF-36 survey, which includes physical and mental health subdomains. Correlations between dominant arm strength and SF-36 subdomain scores were analyzed.

**Results:**

Compared with healthy controls, TDT patients presented significantly lower dominant arm muscle strength (*p* < 0.001). Ferritin levels were elevated (*p* < 0.001), indicating iron overload and chronic anemia. The physical functioning (*p* < 0.001) and physical role difficulty (*p* = 0.002) scores were significantly lower in the TDT group. Handgrip strength was positively correlated with the physical functioning subdomain of SF-36 (*p* = 0.005, *r* = 0.402), while vitality (*p* = 0.009, *r* = 0.379) and mental health (*p* = 0.016, *r* = 0.349) were also associated with overall quality of life scores. No significant correlations were found for emotional or physical role difficulties.

**Conclusion:**

In patients with TDT, reduced handgrip strength was significantly associated with lower health-related quality of life. These findings suggest that handgrip strength may serve as a simple, non-invasive marker of overall well-being in this population. Addressing muscle strength alongside conventional management may help improve both physical and psychosocial outcomes in TDT patients.

## 1 Introduction

Thalassemias, the most common hereditary blood disorders associated with anemia worldwide, exhibit varying regional prevalence rates, with the highest rates observed in Eastern and Southeast Asian countries. According to the 2021 data, the age-adjusted prevalence was reported to be 18.28 per 100,000 individuals, with an incidence of 1.93 per 100,000 individuals (1). *β*-thalassemia is a hereditary blood disorder caused by mutations in the β-globin gene on chromosome 11, resulting in reduced or absent *β*-globin chain synthesis. Patients who require lifelong red blood cell transfusions to maintain pre-transfusion hemoglobin levels within the target range (9.5–10.5 g/dL) are defined as having transfusion-dependent β-thalassemia (β-TDT). This lifelong transfusion requirement remains one of the most significant challenges for patients, as it inevitably leads to transfusion related iron overload and subsequent complications affecting multiple organ systems, including the endocrine, cardiac, hepatic, and musculoskeletal systems (2). As a chronic condition, continuous transfusions can lead to iron overload, resulting in complications and organ dysfunction, particularly affecting the endocrine, cardiac, hepatic, and musculoskeletal systems (3). Musculoskeletal manifestations may include reduced bone density, irregular bone structure, skeletal enlargement, synovial inflammation, joint effusions, vertebral disk abnormalities, spinal irregularities, and growth disturbances of long bones near joints. Although severe bone deformities were common in the pre-transfusion era, they are now rarely observed due to systematic transfusions and the use of modern iron chelators (4). Nevertheless, musculoskeletal impairments such as muscle weakness continue to compromise physical function, leading to fatigue, reduced activity, and decreased productivity, which negatively impact growth, psychosocial well-being, and overall quality of life. Additionally, TDT patients’ psychosocial adjustment and social interactions may be adversely affected by the long-term nature of the disease and ongoing treatment regimen.

At the cellular level, musculoskeletal and organ damage in TDT involves ineffective hematopoiesis and iron overload-induced oxidative stress, which promotes cytokine release and adversely affects myocytes and nerve terminals. Oxidative stress (OS), characterized by increased reactive oxygen species (ROS) and reduced antioxidant defenses, triggers inflammation and damages proteins, lipids, and DNA (5). These processes can result in irreversible nerve and muscle damage, including impaired muscle protein synthesis, atrophy, and reduced myokine production, further weakening motor functions (6). Chronic anemia exacerbates musculoskeletal weakness, reducing muscle strength and endurance, decreasing activity, and diminishing professional performance. The imbalance between muscle synthesis and degradation, combined with anemia-induced inactivity, perpetuates OS-related muscle atrophy and disrupts myokine production, creating a vicious cycle of muscle loss and impaired motor nerve function (7).

Health-related quality of life (HRQoL) assessment is essential in TDT. Studies using the SF-36 have demonstrated reduced physical functioning, emotional well-being, and social engagement among TDT patients compared with healthy controls (8). These challenges, stemming from disease- and treatment-related factors, emphasize the need for comprehensive evaluation of both physical and mental health domains to guide evidence-based interventions aimed at improving quality of life.

This study was based on the hypothesis that reduced handgrip strength, as a marker of muscle weakness, is associated with impaired quality of life in patients with TDT. We anticipated that lower muscle strength would correlate with poorer SF-36 outcomes, providing a simple tool for identifying at-risk patients and guiding interventions to improve both physical function and psychosocial well-being.

## 2 Materials and methods

### 2.1 Study design and participants

This cross-sectional study included 110 participants, comprising 47 patients with *β*-TDT and 63 age and sex matched healthy controls. The study was conducted at the hematology outpatient clinic of a tertiary academic hospital in Turkey, located in an urban area and serving a multi-ethnic population. The sample size was determined by consecutive recruitment of all eligible patients who attended the clinic for routine transfusion visits between January 2024 and December 2024, rather than by an *a priori* power analysis.

Eligible patients were adults (≥18 years) with a confirmed diagnosis of *β*-TDT, receiving regular blood transfusions every 2–4 weeks for at least 1 year. Patients with severe comorbid conditions or additional physical impairments that could independently affect muscle strength or quality of life were excluded; however, individuals with mild or well-controlled co-morbidities commonly observed in TDT were included. Only *β*-TDT patients were enrolled, as no patients with *α*-thalassemia were present in the study population. Healthy controls were recruited from hospital staff and community volunteers, with no known chronic illnesses, and were matched to patients by age and sex. Both patient and control groups were selected to include individuals with comparable levels of routine physical activity (exercise or physiotherapy) and non-labor-intensive occupations, to maintain group homogeneity and minimize potential confounding factors.

All participants provided written informed consent prior to enrollment. The study was conducted in accordance with the principles of the Declaration of Helsinki and was approved by the Ethics Committee of Afyonkarahisar Health Sciences University (approval number: 2023/10, decision dated 10.10.2023).

### 2.2 Outcome measures and data collection

#### 2.2.1 Primary outcome

The primary objective of the study was to evaluate hand grip strength and its impact on quality of life (QoL) and daily productivity. To assess the effect of muscle weakness on work performance, employed participants in both groups were asked to report the total number of workdays missed over the past year, and average annual leave days were calculated for comparison. The association between hand grip strength and QoL in patients with TDT was assessed using the SF-36 health survey.

##### 2.2.1.1 Short Form-36 details

The SF-36 Health Survey was utilized to evaluate the health-related quality of life (HRQoL) of the participants. The Turkish version of the SF-36, validated for reliability and validity (9), was used to assess quality of life in all participants. Given its broad applicability in clinical and research settings, the survey comprehensively evaluates general health outcomes across eight domains: physical functioning (PF), 10 items; general health (GH), 5 items; role-physical (RP), 4 items (which address physical health-related limitations); bodily pain (BP), 2 items; social functioning (SF), 2 items; vitality (VT), 4 items; role-emotional (RE), 3 items (which reflect emotional challenges); and mental health (MH), 5 items (10). Each domain is scored from 0 to 100, with higher scores reflecting better health. Data collected through the SF-36 were analyzed in line with the scoring procedures established by its developers. Its dual focus on physical and mental health ensures its utility as a comprehensive tool for evaluating QoL in clinical populations (11).

#### 2.2.2 Secondary outcomes

The secondary outcomes of this study included the evaluation of additional physical and biochemical parameters potentially associated with muscle strength and quality of life in TDT patients.

The laboratory parameters analyzed were fasting blood glucose (mg/dL), urea (mg/dL), creatinine (mg/dL), alanine aminotransferase (ALT, U/L), aspartate aminotransferase (AST, U/L), sodium (Na, mmol/L), potassium (K, mmol/L), calcium (Ca, mg/dL), thyroid-stimulating hormone (TSH, μIU/mL), ferritin (ng/mL), creatine kinase (CK, U/L), hemoglobin (g/dL), vitamin D (ng/mL), total protein (g/dL), albumin (g/dL), vitamin B12 (pg/mL), folic acid (ng/mL), phosphorus (mg/dL), magnesium (mg/dL), and body mass index (BMI, kg/m^2^). These parameters were selected based on their potential influence on muscle strength and function, as supported by existing literature (12).

##### 2.2.2.1 Handgrip strength

A high-precision digital handgrip dynamometer capable of measuring grip strength up to 396 pounds, with increments of 0.2 lbs. (0.1 kg), was used to assess participants’ muscle strength. Measurements of both right and left hand strength were recorded in kilograms while the participants were at rest, with their upper arms adducted and their elbows positioned at a 90° angle. Participants were classified as having low handgrip strength if their measurements were <28 kg for men and <18 kg for women (13).

### 2.3 Statistical analysis

Categorical variables are presented as percentages and frequencies. The normality of continuous variables was assessed via the Shapiro–Wilk test and visual inspection of histograms. Continuous variables with a normal distribution are expressed as the means and standard deviations, whereas those without a normal distribution are reported as medians and interquartile ranges (IQRs; 25th/75th percentiles). Categorical variables were compared between the groups via the chi-square test. For continuous variables, comparisons were performed via the independent samples *t* test for normally distributed variables and the Mann–Whitney U test for non normally distributed variables. Correlations between dominant arm hand grip strength and SF-36 questionnaire subdomain scores were analyzed via Pearson’s correlation analysis. All *p* values were two-tailed, and values of *p* < 0.05 were considered statistically significant. Statistical analyzes were conducted via SPSS software, version 27.0.1.

## 3 Results

### 3.1 Demographics and clinical characteristics

The study included 110 participants, of whom 47 patients (42.7%) consented to participate from 60 eligible individuals, yielding a response rate of 78%, while the remaining 63 participants (57.3%) comprised the healthy group. Among the patients, 70.2% (*n* = 33) had a history of splenectomy, and 68.1% (*n* = 32) underwent chelation therapy. Some patients did not receive chelation therapy due to recent diagnosis, intolerance, personal preference, or because target ferritin levels had already been achieved. The frequency of erythrocyte suspension transfusion was two units per month in 70.2% of patients (*n* = 33), one unit per month in 21.3% (*n* = 10), and three units per month in 8.5% (*n* = 4). Among all participants, 54.5% (*n* = 60) were female and 45.5% (*n* = 50) were male, with a mean age of 34.41 ± 3.8 years. Age and sex distributions were similar between the patient and control groups (*p* = 0.122) (Table 1).

**Table 1:** Comparison of demographic and laboratory parameters between the patient and control groups.

Characteristic	Patient (*n* = 47)	Control (*n* = 63)	*p*
Age (years)	34.05 ± 2.9	33.5 ± 2.2	0.569
Female gender, % (*n*)	63.8 (30)	47.6 (30)	0.122
Active employment, % (*n*)	38.3 (18)	76.2 (48)	**<0.001**
BMI (kg/m^2^)	23.3 (21.2–23.5)	24.2 (20.9–28.1)	0.171
Glucose (mg/dL)	93 (84–110)	90 (86–108)	0.758
Urea (mg/dL)	27 (21–34)	26.3 (20.9–34.1)	0.648
Creatinine (mg/dL)	0.57 (0.49–0.69)	0.5 (0.5–0.6)	0.350
AST (U/L)	28 (21–39)	27.4 (20.9–32.5)	0.476
ALT (U/L)	22 (13–42)	27 (20.9–34.6)	0.176
Sodium (mmol/L)	139 (137–140)	140 (138–141)	0.198
Potassium (mmol/L)	4.4 (4.2–4.7)	4.2 (3.8–4.5)	0.336
Phosphorus (mmol/L)	4.1 (3.8–4.46)	4 (3.8–4.1)	0.157
Magnesium (mmol/L)	2 (0.86–2.16)	2.4 (2.2–3)	0.235
Calcium (mmol/L)	9.44 (9.1–9.67)	9.6 (9.5–9.6)	0.234
Ferritin (ng/mL),	1,343 (774–1,650)	30 (21.4–32.5)	**<0.001**
Vitamin B12 (pg/mL),	374 (293–459)	331 (265–370)	0.266
Folic acid (pg/mL),	6.41 (4.8–17)	6.8 (4.8–8.9)	0.225
Vitamin D (ng/mL),	25.7 (10–23.6)	26 (22–33)	0.905
TSH (μIU/mL)	2.79 (1.76–3.81)	3.1 (2.1–3.8)	0.527
Hemoglobin (Hb) (g/dL)	8.5 (7.8–9.5)	13.8 (13.2–14.1)	**<0.001**
Creatine kinase (U/L)	26 (18–29)	29 (25–44)	0.368
Total protein (g/dL)	7.4 (7–7.7)	7.14 (6.7–7.7)	0.201
Albumin (g/dL)	4.7 (4.4–5.14)	4.74 (3.2–4.1)	0.889

Bold values indicate statistical significance (*p* < 0.05).

BMI and biochemical markers, including glucose, urea, creatinine, AST, and ALT, were comparable between the groups. The number of actively working employees differed significantly, with patients using an average of 22 leave days per year and controls using an average of 10 days (*p* < 0.001). Ferritin levels were significantly higher in the patient group (*p* < 0.001), while hemoglobin levels were significantly lower (*p* < 0.001). Electrolyte and mineral levels, including sodium, potassium, phosphorus, magnesium, and calcium, as well as vitamin D, vitamin B12, folic acid, and TSH, were similar between groups (*p* > 0.05), (Table 1).

### 3.2 SF-36 scores

Analysis of the SF-36 survey revealed differences between patient and control groups in several domains. Physical function scores were lower in patients (80 [50–90]) than controls (100 [90–100], *p* < 0.001), and physical role difficulty scores were also reduced in patients (50 [25–100] vs. 100 [50–100], *p* = 0.002). Emotional role difficulty (66.7 [33.3–100] vs. 66.7 [0–100], *p* = 0.054), energy/vitality (55 [40–65] vs. 50 [40–70], *p* = 0.794), mental health (64 [48–76] vs. 56 [48–76], *p* = 0.371), social functioning (75 [50–87.5] vs. 75 [50–87.5], *p* = 0.909), and pain (67.5 [45–90] vs. 77.5 [57.5–90], *p* = 0.166) did not differ significantly between groups. General health scores were lower in patients (50 [40–60] vs. 60 [45–70], *p* = 0.005). The Physical Component Summary (PCS) was lower in patients (65.2 ± 12.3) than controls (82.5 ± 10.4, *p* < 0.001), whereas the Mental Component Summary (MCS) did not differ significantly (70.1 ± 11.8 vs. 73.4 ± 9.7, *p* = 0.148). A comparison of the SF-36 survey subscales and summary scores between the patient and control groups is shown in Table 2.

**Table 2:** Comparison of SF-36 survey subscales between patient and control groups.

Survey subscale	Patient (*n* = 47)	Control (*n* = 63)	*p*
Physical function	80 (50–90)	100 (90–100)	<0.001
Physical role difficulty	50 (25–100)	100 (50–100)	0.002
Emotional role difficulty	66.7 (33.3–100)	66.7 (0–100)	0.054
Energy, vitality	55 (40–65)	50 (40–70)	0.794
Mental health	64 (48–76)	56 (48–76)	0.371
Social functioning	75 (50–87.5)	75 (50–87.5)	0.909
Pain	67.5 (45–90)	77.5 (57.5–90)	0.166
General health	50 (40–60)	60 (45–70)	0.005
Physical component summary (PCS)	65.2 ± 12.3	82.5 ± 10.4	<0.001
Mental component summary (MCS)	70.1 ± 11.8	73.4 ± 9.7	0.148

### 3.3 Dominant muscle strength

**Figure 1:**
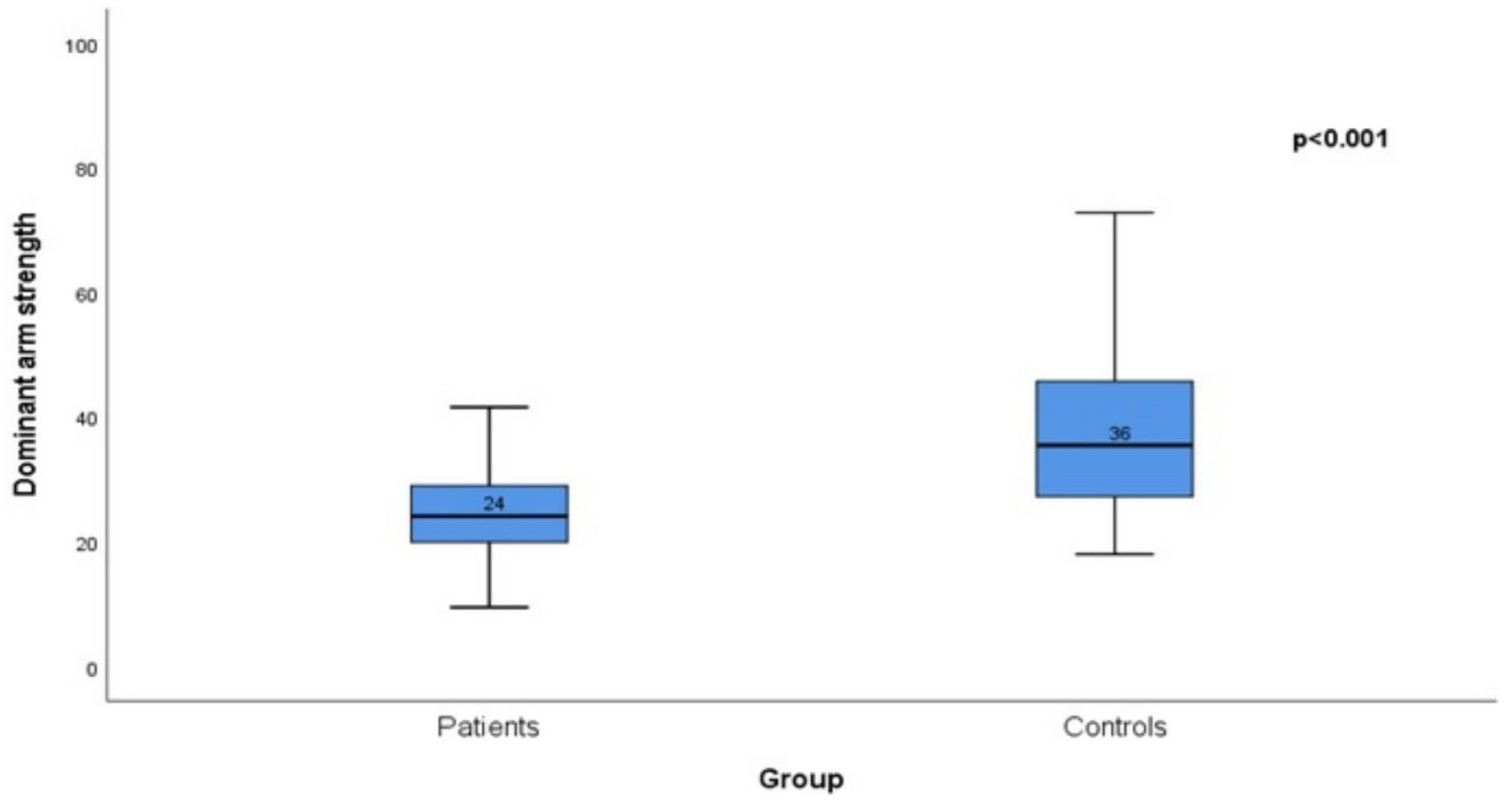
Comparison of dominant arm strength (kg) between the patient and control groups.

The median dominant arm strength of the patient group was significantly lower than that of the control group (*p* < 0.001; Figure 1).

### 3.4 Correlations

**Figure 2:**
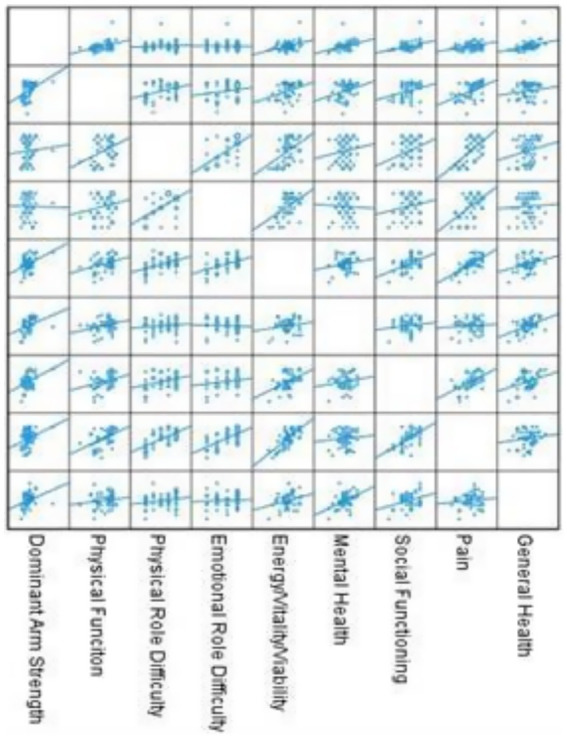
Correlations between dominant hand grip strength and the SF-36 subscale.

In the patient group, dominant arm hand grip strength was significantly correlated with several SF-36 subscales: physical function (*p* = 0.005, *r* = 0.402), energy/vitality (*p* = 0.009, *r* = 0.379), mental health (*p* = 0.016, *r* = 0.349), social functioning (*p* = 0.014, *r* = 0.357), pain (*p* = 0.038, *r* = 0.304), and general health (*p* = 0.033, *r* = 0.311; Figure 2). No significant correlation was observed with physical role difficulty (*p* = 0.644) or emotional role difficulty (*p* = 0.926).

Figure 2 illustrates the correlation between the scores of the subscales of the SF-36 questionnaire and dominant arm hand grip strengthin the patient group.

## 4 Discussion

This study aimed to comprehensively evaluate the association between hand grip strength and quality of life in patients with TDT using the SF-36 quality of life scale. The findings indicated that lower handgrip strength in this patient group was associated with decreased scores across several SF-36 subdomains, particularly physical functioning, vitality, and mental health, highlighting the negative impact of muscle weakness on overall quality of life. Considering the mean age of the participants (34.41 ± 3.8 years), the effects of chronic disease-related physical and psychosocial burdens on the quality of life of middle-aged patients with TDT became more evident, with physical fatigue and reduced muscle strength significantly contributing to diminished well-being. Notably, advanced age plays a critical role in meeting daily needs and in participating more actively in social life. In patients with TDT, advancing age often brings increasing life responsibilities, which may exacerbate the physical and psychosocial challenges associated with the disease and potentially affect quality of life. Although age-related declines in overall health have been reported in the literature, this study did not specifically assess the impact of age on SF-36 scores (14–16).

The fact that 70.2% of the patients in the patient group had a history of splenectomy and that the majority (68.1%) were receiving oral iron ICT highlights their high risk of complications (Table 1). Additionally, the proportion of patients receiving two units of red blood cell suspension per month (70.2%) underscores the fundamental role of transfusion in maintaining hematological control and managing complications in individuals diagnosed with TM. The impact of transfusion frequency and ICT on quality of life has also been a prominent topic of discussion in recent studies (17). Effective patient centered treatments are needed to incorporate these individuals into a healthier life process. While treatment adherence and anemia management play major roles in improving physical performance, ICT is critical in mitigating inflammation and oxidative processes. Regular blood transfusions and effective ICT can alleviate some of these deficiencies. However, adherence remains a challenge, particularly in resource-limited regions. The lower scores observed in these areas further demonstrate this issue (18). Correcting anemia in TDT patients with approved treatments, including non-transfusion-based therapies such as gene therapy and supportive options such as luspatercept, has the potential to reduce the burden of RBC transfusions, improve clinical outcomes, and increase quality of life (19).

The patient group showed significantly lower hemoglobin levels and markedly higher ferritin levels compared with the control group, whereas other biochemical markers, such as total protein, albumin, and creatine kinase, did not differ significantly (Table 1). Although our study did not directly evaluate the effects of hemoglobin and ferritin levels on quality of life, the observed low hemoglobin and high ferritin in TDT patients provide context for interpreting the association between reduced hand grip strength and physical functioning. These hematological parameters may contribute to suboptimal oxygen delivery and potential iron-related oxidative stress, which can influence muscle performance and overall well-being. Thus, our findings support the relevance of careful hematological management in maintaining physical function and quality of life in TDT patients, consistent with previous literature (20, 21).

The positive relationships between hand grip strength and quality of life subdomains such as physical functioning, energy/vitality, social functioning, and mental health suggest that muscle performance contributes not only to physical well-being but also to psychosocial well-being. This finding underscores the potential of muscle-strengthening interventions to improve the quality of life of TDT patients. However, no significant relationship was found between hand grip strength hand the subdomains of physical and emotional difficulties. These results indicate that these dimensions may be linked not only to physical capacity but also to the psychosocial and emotional aspects of the disease. In particular, the emotional and social burdens of patients may further complicate the impact of their condition on their daily functionality. Enhanced hand grip strength appears to have positive effects on both physical and psychosocial well-being (22, 23). For example, in a study conducted on patients with fibromyalgia syndrome, a pooled exercise program improved the mental health scores of the SF-36 and reduced patient complaints. This finding indicates that reducing muscle weakness can enhance quality of life. Similarly, another study conducted on stroke patients revealed that sensory and motor problems in the upper extremities had a significant effect on quality of life, as measured by the SF-36. This finding shows that muscle weakness limits an individual’s activities of daily living and social participation, thereby reducing their quality of life. The results of our study revealed that reduced hand grip strength is associated with lower quality of life, as measured by the SF-36 questionnaire, and this relationship is consistent across different patient groups. Therefore, preserving and enhancing hand grip strength play critical roles in improving the quality of life of individuals (24–26). The significant differences in clinical characteristics between the patient and control groups, including anemia and iron overload, suggest that physical fatigue and reduced muscle strength may be major contributing factors (Table 1).

Free iron accumulation may promote inflammation and muscle breakdown, impairing physical performance and daily functioning, while damage to key organs can further affect quality of life; iron chelation therapy is important for mitigating these effects (5). These findings emphasize the importance of not only regular blood transfusions and ICT in the management of TDT but also carefully addressing the side effects of these treatments (27). The patient group had significantly lower scores than the control group in physical functioning and physical role domains. Additionally, significant differences were observed in general health perception and social functioning (*p* < 0.001) (Table 2; Figure 1). Similar to findings from other studies that highlight significant impairments in physical health parameters among patients with TDT, our results indicate a direct impact on quality of life. A meta-analysis conducted using the SF-36 questionnaire in patients with *β*-TM demonstrated that subdomains related to physical health were significantly lower than those in the general population. This meta-analysis underscores the substantial impact of TDT on physical health, which adversely affects quality of life (8, 28, 29). Although there was no significant difference between the groups in the pain subscale (*p* = 0.166), the control group scored higher on the general health perception subscale (*p* = 0.005). The long-term treatment processes and complications of TDT negatively affect patients’ perceptions of their general health. The literature also indicates that individuals with chronic illnesses often report lower health perceptions (30). Additionally, there was no significant difference between the two groups in terms of emotional role difficulty (*p* = 0.054). The similar emotional functioning scores between the TDT patients and the control group suggest that these individuals may have developed emotional resilience. However, the wider distribution of scores in the patient group indicated that some individuals may face greater emotional challenges. In addition to these evaluations, this public health issue has a socioeconomic dimension. Unfortunately, most of these patients are unable to work and rely on state and family support for their livelihoods. Only 38% of our patients were actively employed. Among those employed, the average annual sick leave was 22 days, which corresponds to the time typically required for regular bi-monthly transfusions in TDT patients. These findings illustrate the expected effect of treatment schedules on work attendance in this population (31).

The lack of access to appropriate rehabilitation treatments in the early stages for these patients is associated with significant health issues and workforce loss. During inactivity, a vicious cycle occurs, as muscle hypotrophy develops (32). In the short term, fatigue symptoms can be alleviated through individualized treatment and supportive interventions. In the long term, effective approaches include promoting gene therapy, one of the most effective methods for eliminating the need for transfusions, along with optimized ICT protocols that address adherence issues. Establishing centers that provide comprehensive physical and psychological rehabilitation involving hematologists, physiotherapists, die-titians, and psychologists is crucial. Rehabilitation programs aimed at improving physical performance and psychosocial support initiatives to enhance general health perception are crucial for TDT patients. Additionally, providing education and counseling services to patients and their families could help support their emotional functioning. TDT is a serious public health issue that imposes a substantial economic burden. From this perspective, innovative approaches, such as multidisciplinary care, may help improve or enhance the quality of life of TDT patients.

The limitations of this study are as follows: This study included a limited number of participants. Conducting similar studies in larger populations would increase the reliability of the results. Additionally, the cross-sectional design of this study made it difficult to establish causal relationships. A prospective evaluation of patients’ hand grip strength and SF-36 results following a decrease in ferritin levels due to iron chelation therapy could provide a more detailed observation of the effects of treatment on quality of life. Although dominant arm hand grip strength was assessed, the strength and functionality of other muscle groups (e.g., legs or back) could not be evaluated owing to technical limitations. Ferritin levels have been used as indicators of iron accumulation; however, direct measurements of tissue iron levels have not been performed. Addressing these limitations explicitly can provide recommendations for future studies and enhance the contextual strength of our findings.

## 5 Conclusion

This study demonstrates that patients with TDT have reduced hand grip strength compared to healthy controls, which is associated with lower scores in several domains of quality of life as assessed by the SF-36. The findings indicate that reduced hand grip strength compromises physical function and may also be associated with psychosocial well-being, highlighting the potential role of muscle weakness in affecting physical functioning and general well-being. While hand grip strength showed positive correlations with certain quality-of-life subdomains, some dimensions, including emotional and physical role difficulties, were not significantly associated, emphasizing the multidimensional nature of the disease. Factors including chronic transfusions, iron overload, and older age can contribute to increased physical fatigue, oxidative stress, and systemic inflammation, potentially diminishing quality of life in TDT patients.

These results suggest that interventions targeting muscle strength, along with comprehensive management of TDT including optimized iron chelation and multidisciplinary support—may contribute to improved physical outcomes and potentially enhance psychosocial well-being, although causality cannot be confirmed due to the observational design and possible confounding factors. Future studies should further investigate how clinical characteristics, such as adherence to iron chelation therapy, degree of iron overload, and comorbidities, influence hand grip strength and quality of life in this patient population.

## Data Availability

The datasets presented in this study can be found in online repositories. The names of the repository/repositories and accession number(s) can be found in the article/supplementary material.
